# Natriuretic peptide receptor C in cardiovascular–kidney–liver–metabolic syndrome: from natriuretic peptide deficiency to direct tissue signaling?

**DOI:** 10.1007/s11154-026-10066-1

**Published:** 2026-07-10

**Authors:** Matteo Landolfo, Francesco Spannella, Chiara Salvà, Bernhard Radlinger, Susanne Kaser, Alessandro Gezzi, Riccardo Sarzani

**Affiliations:** 1 Department of Internal Medicine I, Medical University of Innsbruck, Innsbruck, 6020 Austria; 2 Clinical and Molecular Sciences Department, University Politecnica delle Marche, Ancona, 60126 Italy; 3 Clinical Medicine and Geriatrics, IRCCS INRCA, Ancona, 60126 Italy

**Keywords:** Cardiometabolic medicine, Natriuretic peptide receptor C, *Npr3*, C-type natriuretic peptide, Musclin, Osteocrin, C-ANP_4−23_

## Abstract

The cardiovascular–renal–liver–metabolic (CKLM) syndrome integrates dysmetabolically driven heart, vascular, kidney, and liver diseases through a shared pathophysiological substrate. In obesity, upregulation of the clearance natriuretic peptide receptor C (NPR-C) in adipose tissue creates a “NP deficiency,” undermining the protective cardiovascular and metabolic actions of endogenous NPs. Recent preclinical data demonstrates that NPR-C functions beyond simple peptide clearance. Through interactions with ligands like CNP, musclin, and osteocrin, NPR-C triggers context-dependent intracellular signaling. In experimental models, independent of systemic NP levels, NPR-C directly modulates cardiac remodeling, podocyte injury, hepatic steatosis, vascular inflammation, and adipocyte function. This review synthesizes NPR-C biology within the CKLM framework. While human validation remains limited, targeting tissue-specific NPR-C pathways represents a promising therapeutic frontier for restoring cardiometabolic homeostasis.

**Graphical Abstract:**

**Figure:**
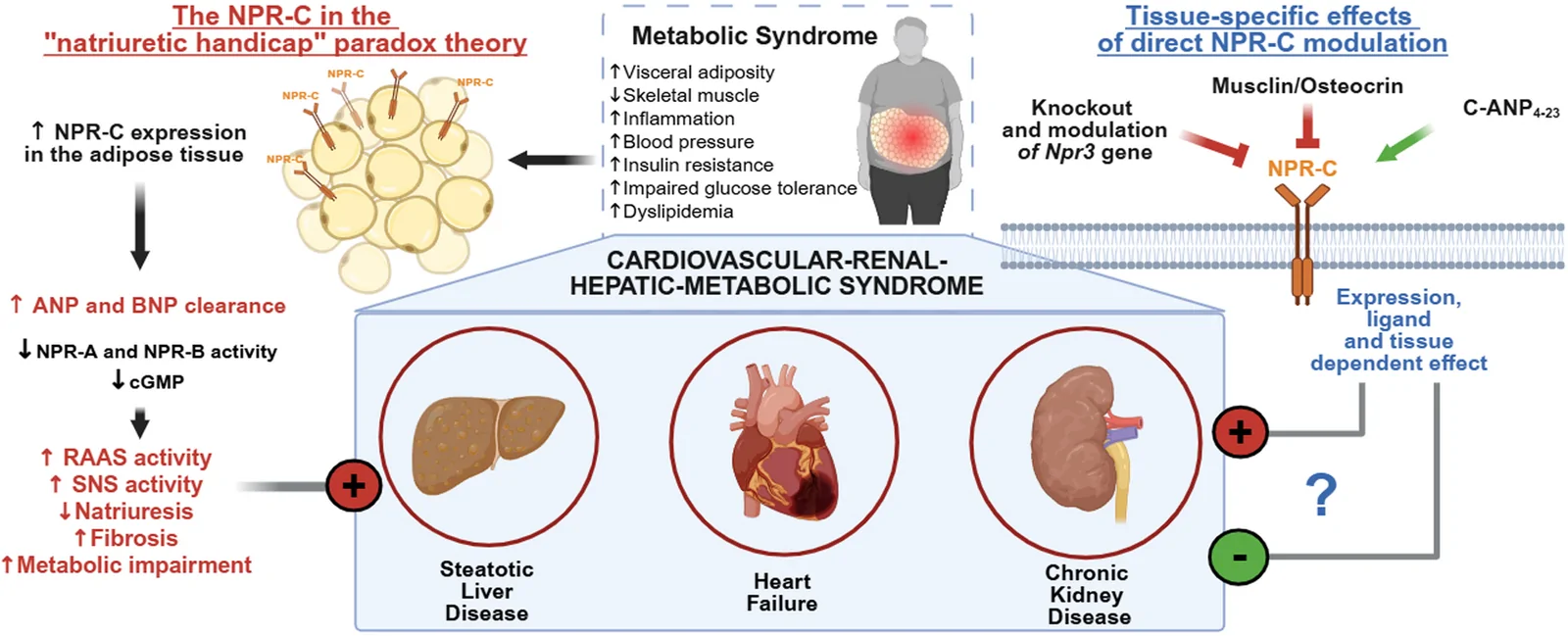

## Introduction

The cardiovascular-kidney-metabolic (CKM) syndrome provides a unifying framework for understanding the complex multisystem interactions linking dysmetabolically driven heart failure (HF), chronic kidney disease (CKD), and metabolic dysfunction [1]. Emerging evidence supports extending this paradigm to include metabolic dysfunction–associated steatotic liver disease (MASLD), giving rise to the concept of cardiovascular-kidney-liver-metabolic (CKLM) syndrome. This expanded framework is supported by growing epidemiological and clinical evidence demonstrating an increased risk of cardiovascular disease (CVD) among individuals with MASLD [2, 3], as well as by a shared pathophysiological substrate characterized by excess visceral adiposity, hyperinsulinemia, insulin resistance, and chronic low-grade inflammation [4, 5]. Together, these interconnected mechanisms drive the development and progression of cardiovascular, renal, hepatic, and metabolic dysfunction while influencing disease trajectories and therapeutic responsiveness across these organ systems [6]. Consequently, the CKLM framework has important implications for disease screening, risk stratification, staging, and therapeutic decision-making, shifting the focus from single-organ disease toward integrated cardiometabolic and multiorgan risk modification.

Among the biological systems linking these interconnected organs, the natriuretic peptide (NP) network has emerged as a key regulatory axis. Atrial, B-type, and C-type natriuretic peptides (ANP, BNP, and CNP), produced primarily by the myocardium and vascular endothelium, exert pleiotropic cardiovascular, renal, hepatic, and metabolic effects through activation of the guanylyl cyclase–coupled receptors NPR-A and NPR-B. The resulting cyclic guanosine monophosphate (cGMP)-dependent signaling promotes vasodilation, natriuresis, antifibrotic activity, and favorable metabolic adaptations, including enhanced lipolysis, mitochondrial oxidative metabolism, and energy expenditure [7–9].

In contrast, natriuretic peptide receptor-C (NPR-C) has traditionally been viewed primarily as a clearance receptor, mediating receptor-dependent internalization and degradation of circulating NPs. Although limited intracellular signaling through inhibitory G protein (G_*i*_)-coupled pathways has been described, its physiological significance in humans remains incompletely defined [10, 11].

Nevertheless, accumulating evidence from experimental studies, animal models, and human genetic and clinical investigations has repositioned NPR-C as a node in the regulation of NP bioavailability and cardiometabolic homeostasis [12].

Dysregulation of NPR-C expression and function has been linked to obesity, insulin resistance, hypertension, adverse cardiovascular remodeling, and other manifestations of metabolic dysfunction.

Given its ability to modulate NP signaling at the interface of cardiovascular, renal, hepatic, vascular, and metabolic pathways, NPR-C may represent a mechanistic node within the emerging CKLM framework. In this review, we synthesize current evidence regarding the role of NPR-C across the cardiovascular, kidney, liver, and metabolic continuum, highlighting its contribution to disease pathophysiology and evaluating its potential as a therapeutic target in this increasingly prevalent and clinically complex syndrome.

## NPR-C biology in humans

In humans NPR-C, encoded by the natriuretic peptide receptor 3 (*NPR3*) gene, is the most abundantly expressed and widely distributed member of the NPR family. It is found across various endocrine organs, the kidneys, adipose tissue and the lungs, with particularly high expression levels localized in endothelial cells [13, 14].

Structurally, NPR-C is a single-pass transmembrane protein that shares significant homology with the receptors NPR-A and NPR-B. However, NPR-C lacks an intracellular guanylyl cyclase domain. Consequently, it does not generate cGMP upon ligand binding and canonically acts as a clearance receptor, serving as a regulator of both systemic and local ANP and BNP bioavailability [11]. In addition to mediating ligand internalization, NPR-C negatively regulates NP signaling by forming non-functional heterodimers with NPR-A and NPR-B. This direct membrane interaction physically prevents cGMP synthesis, expanding the known inhibitory repertoire of NPR-C [15, 16].

Compelling evidence from human studies supports the physiological clearance function of NPR-C, a role that is most clearly illustrated by the phenotypic consequences associated with NPR-C deficiency. A global absence of NPR-C leads to a significant extension of the plasma half-life of ANP, confirming that NPR-C is a dominant pathway for its systemic removal. Interestingly, this does not hold true for BNP, whose half-life remains largely unaffected by the absence of the receptor. This discrepancy suggests that while ANP is primarily receptor-cleared, BNP may rely more heavily on other degradative pathways, such as enzymatic cleavage by neprilysin [14, 17].

From the clinical perspective, biallelic loss-of-function mutations in the NPR-C gene other than causing a distinct overgrowth syndrome characterized by tall stature, long fingers, joint hyperlaxity, and aortic dilatation, leads to the systemic overactivation of the NPR-A and NPR-B pathway by cardiac- and endothelial-derived NPs. This overactivation triggers increased adipocyte browning, suppressed renin-aldosterone release, and enhanced vasodilation, a mechanism supported by the elevated plasma cGMP and altered pro-peptide ratios; therefore, affected individuals also exhibit low blood pressure (BP) and reduced overall adiposity [18, 19]. Population-based and Mendelian randomization studies further implicate NPR-C gene variants in the modulation of BP regulation, hypertension susceptibility, and risk of cardiovascular disease, underscoring the pleiotropic roles of NPR-C in human pathophysiology [20, 21].

## Human evidence for NPR-C-mediated NP deficiency in obesity

**Fig. 1:**
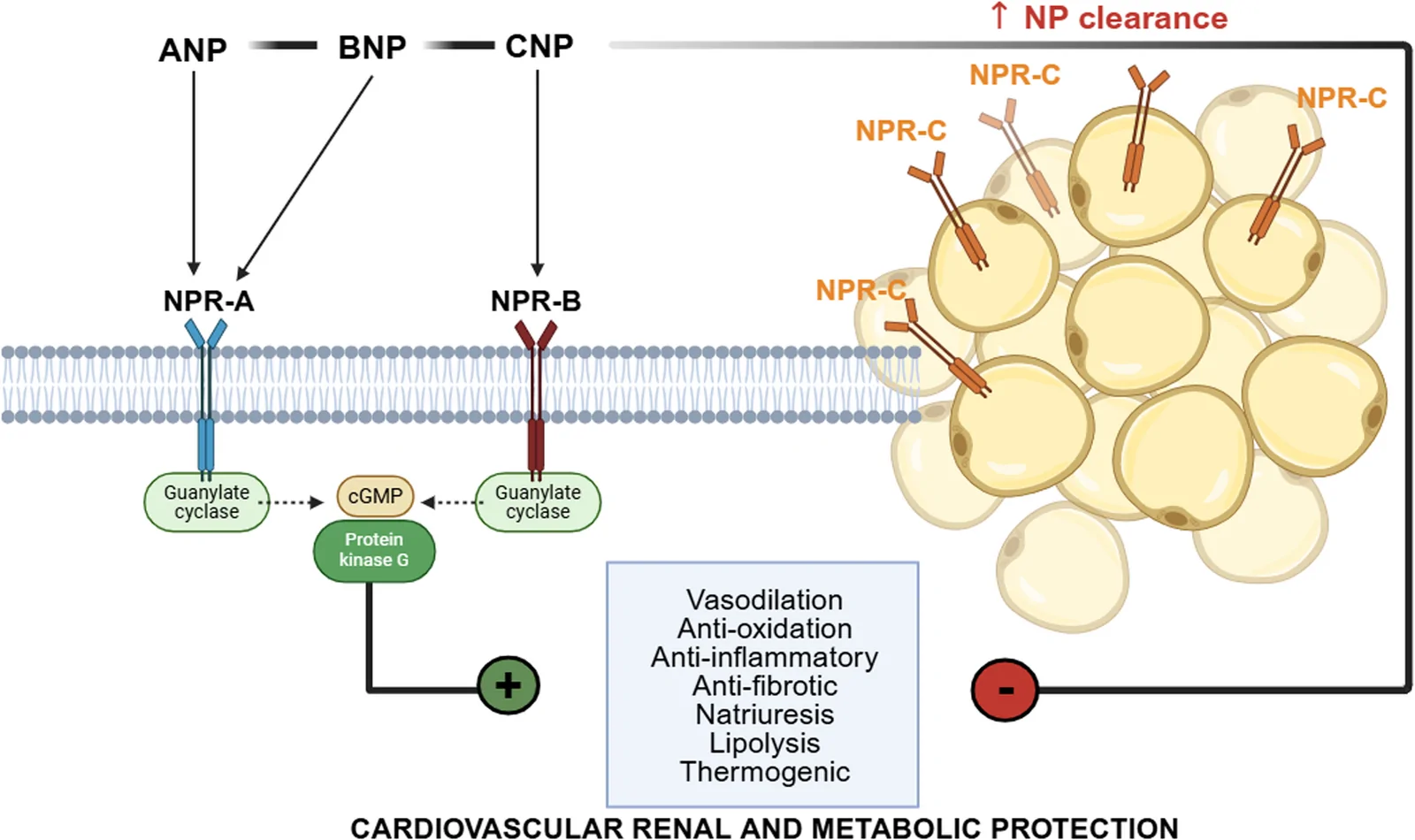
The role of NPR-C in the natriuretic peptide deficiency paradox theory. On the left, atrial natriuretic peptide (ANP) B-type and C-type natriuretic peptide (BNP and CNP) bind to NPR-A and NPR-B receptors, activating a signaling cascade promoting cardiovascular, renal and metabolic protective effects. Conversely, on the right, the increased expression of the NPR-C clearance receptors in metabolically dysfunctional adipose tissue, reduces NP bioavailability and their local and systemic beneficial effects

Obesity and metabolic dysfunction, driven by hyperinsulinemia and insulin resistance, represent central pathophysiological drivers of CKLM, linking excess visceral adiposity to multisystem organ injury. Yet, despite this clear mechanistic role, a fundamental paradox exists in clinical obesity: despite the presence of cardiometabolic stressors that should stimulate NP production, circulating NP levels remain consistently low [22–24]. Highlighting this paradox, a recent analysis of two trials in patients with HF with reduced ejection fraction showed that greater adiposity was linked to higher aldosterone levels but lower BNP concentrations [25]. Similarly, in patients with HF with preserved ejection fraction (HFpEF), which is the most prevalent phenotype among patients with obesity, NPs levels were inversely correlated with BMI [26, 27]. This phenomenon is well-supported by specific studies on human obesity, which identify the upregulation of NPR-C in adipose tissue as the primary driver (Fig. 1).

Notably, visceral and subcutaneous adipose tissue from patients with obesity exhibit elevated NPR-C expression, which correlates positively with the degree of adipocyte hypertrophy and the severity of insulin resistance [28]. Also, data show a relative increase of adipose NPR-C expression (and decreased NPR-A/NPR-C ratio) in individuals with obesity and insulin resistance, with NPR-C levels correlating with fasting insulin and metabolic risk markers [29]. Experimental studies in human-derived mature adipocytes, showed NPR-C expression to increase following exposure to incremental concentrations of insulin and glucose [30].

However, beyond increased NPR-C expression, multiple complementary mechanisms have been implicated in the NP deficiency in this setting, including altered NP processing and glycosylation, obesity-related cardiac remodeling with impaired NP secretion, reduced physical activity, and complex hormonal and metabolic perturbations [31]. Consequently, the lower circulating NP concentrations observed in obesity are now thought to reflect the combined effects of altered peptide production, processing, secretion, and clearance. Still, the relative contribution of each mechanism remains incompletely understood.

Given that NPR-C is a determinant of the strength and duration of NP signaling, the relative reduction in biologically active NP signaling impairs their vasodilatory, natriuretic, antifibrotic, lipolytic, and thermogenic actions. As a result, pathological vascular and cardiac remodeling, renal sodium retention, dyslipidemia, and impaired energy metabolism are exacerbated, contributing to increased risk of hypertension, type 2 diabetes mellitus (T2DM), and HF in this population [29, 32, 33].

## Preclinical evidence for direct NPR-C signaling

Available human evidence largely supports a predominant role for NPR-C as a clearance receptor, particularly in cardiovascular and metabolic tissues where regulation of NP availability appears to be its principal physiological function. By contrast, the concept of NPR-C as an active signaling receptor is supported mainly by experimental data from cellular and animal models, while direct evidence in humans remains scarce. In these settings, 2 subtypes of NPR-C have been reported: one with high affinity for CNP, in which the short intracellular domain of has been shown to couple to G_*i*_ proteins, leading to inhibition of adenylyl cyclase, activation of phospholipase C, stimulation of endothelial nitric oxide synthase (eNOS), and modulation of MAPK signaling pathways, and a low-affinity receptor involved in ligand internalization [34]. Whether these signaling properties substantially contribute to human physiology and disease beyond NPR-C-mediated NP clearance remains an area of ongoing investigation [10, 35, 36].

Furthermore, in vitro investigations utilizing physiological and pharmacological ligands with varying receptor modulatory activities have elucidated the intracellular signaling pathways coupled to NPR-C. For instance, CNP—traditionally viewed as an NPR‐B ligand—exerts potent local paracrine and autocrine effects via NPR‐C interaction, particularly within the vascular endothelium and myocardium [14, 37–41].

Beside CNP, osteocrin (or musclin)—secreted primarily by skeletal muscle and bone—functions as a high-affinity endogenous antagonist for NPR-C. By competitively binding to the NPR-C, osteocrin limits the clearance of NPs, effectively boosting NPR-A and NPR-B signaling in target tissues such as the myocardium, the central nervous system, and the musculoskeletal system [42–47].

C-ANP_4−23_, is commonly used as an NPR-C-selective ligand. Depending on the experimental context, it may activate NPR-C–G_*i*_ signaling while also competitively reducing endogenous NP clearance, thereby functionally increasing NPR-A/NPR-B ligand availability [48, 49].

Another selective NPR-C antagonist (M372049) was used in animal models to confirm the contribution of CNP/NPR-C signalling in the regulation of arterial vascular tone and investigate the mechanism(s) underlying the release and biological activity of CNP at the endothelium level [38].

Further modulation of the NPR-C was described in studies designed to assess the effect of miRNAs (to date, miR-100, miR-143 and miR-146a) on its activity by modulating *Npr3* expression [50–52].

Far from being a simple sink for circulating NPs, NPR-C is part of a finely tuned ligand–receptor network, encompassing osteocrin/musclin, that dictates local peptide gradients, the spatial compartmentalization of cGMP signaling, and nuanced physiological responses across various tissues.

## Direct NPR-C modulation within the CKLM syndrome components

The following sections examine the effects of NPR-C expression and function across the individual components of the CKLM spectrum, spanning metabolic dysfunction and obesity to established cardiovascular, renal, and hepatic organ damage. Particular emphasis is placed on the strength and translational relevance of the available evidence, distinguishing findings derived from clinical studies from those originating in experimental models. Importantly, current mechanistic understanding of NPR-C regulation and function remains limited, as much of the evidence is derived from in vitro systems and animal studies. Moreover, substantial heterogeneity in experimental designs, disease models, and methods of NPR-C modulation complicates the interpretation and comparison of results, thereby limiting their direct applicability to human physiology. Consequently, many of the proposed mechanisms and therapeutic implications require further validation in well-designed clinical and translational studies. Each subsection follows a structure based on a hierarchical level of evidence, from human studies to tissue or cellular models, passing through preclinical in vivo models.

The putative effects of NPR-C modulation across the various experimental models are summarized in Table 1.

**Table 1:** Overview of NPR-C modulation across experimental models of the CKLM syndrome components

Organ System	Model	NPR-C Modulation	Key Findings	Net Pathophysiological Outcome
Heart	Murine Ang II-induced hypertensive heart disease	Global *Npr3* knockout	NPR-C^−/−^ mice treated with Ang II showed aggravated decline in cardiac function and accelerated decompensation into overt HF compared to wild-type [53]	Harmful effect of NPR-C deletion
	Murine Ang II-induced atrial remodeling	C-ANP_4−23_ treatment	C-ANP_4−23_ reduced AF susceptibility by preventing most of the Ang II–induced atrial myocardial abnormalities [54]	Beneficial effect of NPR-C inhibition
	Isolated human cardiac fibroblasts	C-ANP_4−23_ treatment	NPR-C antagonism blocked BNP-mediated inhibition of proliferation of human cardiac fibroblasts [55]	Harmful effect of NPR-C inhibition
	Murine diabetic cardiomyopathy models	Global *Npr3* knockout	Attenuated myocardial fibrosis and preserved ventricular function via PKA/PKG activation and suppression of TGF-β/Smad signaling [56]	Beneficial effect of NPR-C deletion
	Murine atrial myocardium	Tissue-specific *Npr3* knockout	Attenuation of TGF-β1-induced interstitial fibrosis and electrical derangement [57]	Beneficial effect of NPR-C deletion
	Murine MI and pressure-overload models	Osteocrin overexpression or administration	Increased NP bioavailability, attenuated adverse remodeling, reduced hypertrophy and ventricular dilation [58, 59]	Beneficial effect of NPR-C inhibition
	Murine acute cardiac ischemia	C-ANP_4−23_ treatment	C-ANP_4–23_ promoted coronary vasodilation, limited infarct size, and preserved post-ischemic contractility [60]	Beneficial effects of NPR-C inhibition
	Cardiomyocyte-specific CNP models	Local CNP/NPR-C modulation	Enhanced myocardial revascularization and minimized ischemic injury and reactive fibrosis [61]	Beneficial effect of NPR-C activation
Kidney	FSGS and diabetic kidney disease models	Podocyte-specific *Npr3* knockout	Ameliorated albuminuria, preserved podocyte structural integrity, and reduced expression of inflammatory/pro-fibrotic genes [62–64]	Beneficial effect of NPR-C deletion
	Hypertensive and glomerular injury models	C-ANP_4−23_ treatment	Attenuated proteinuria and renal fibrosis, reduced podocyte stress via enhanced local NP availability [49, 64]	Beneficial effect of NPR-C inhibition
	Isolated distal tubule cells	*Npr3* deletion	Downregulated NaCl cotransporter activity, protecting against Ang II-induced systemic hypertension [65]	Beneficial effect of NPR-C deletion
Adipose tissue and muscle	Diet-induced obese mice	Global *Npr3* knockout Adipose tissue *Npr3* deletion	Promoted white adipose tissue browning, boosted energy expenditure/thermogenesis, and conferred resistance to diet-induced obesity [66–69]	Beneficial effect of NPR-C deletion
	Murine hypercholesterolemic models	Global *Npr3* knockout	Attenuated chronic adipose tissue inflammation and reduced macrophage infiltration [67].	Beneficial effect of NPR-C deletion
	Murine adipose tissue and human SGBS cells	miR-146a modulation of *Npr3*	miR-146a downregulates *Npr3*; its inhibition reduced insulin-stimulated glucose uptake, whereas while transfecting miR-146a restored metabolic profiles [52].	Beneficial effect of NPR-C downregulation
	Murine skeletal muscle cells	Musclin administration	Impaired insulin receptor signaling and downstream metabolic responses in skeletal muscle [70]	Harmful effect of NPR-C inhibition
	Murine adipose tissue in high-fat diet	Musclin administration	Restricted diet-induced visceral fat accumulation in both sexes [71]	Beneficial effect of NPR-C inhibition
	3T3-L1 murine adipocytes	Musclin administration	Augmented lipolytic pathways while concurrently suppressing de novo lipogenesis [72]	Beneficial effect of NPR-C inhibition
	Murine leptin-deficient (*ob/ob)* obese models	C-ANP_4−23_ treatment	Reduced total adiposity and improved peripheral insulin sensitivity [73]	Beneficial effect of NPR-C inhibition
Vascular System	SHR and Dahl salt-sensitive rats	C-ANP_4−23_ treatment	Reduced systemic BP, attenuated vascular oxidative stress, and minimized endothelial injury [74]	Beneficial effect of NPR-C inhibition
	Spontaneous hypertensive rats	Musclin administration	Exacerbated systemic hypertension, mediated in part through direct NPR-C binding interactions [75]	Potentially harmful effect of NPR-C inhibition
	Isolated murine resistance arteries	CNP stimulation ± M372049 block	Evoked vasorelaxation via an NPR-C/G_*i*_-dependent pathway coupled with EDHF hyperpolarization [38]	Beneficial effect of NPR-C activation
	Vascular smooth muscle cells	C-ANP_4−23_ treatment	Modulated abnormal vascular smooth muscle cell proliferation and migration kinetics [76, 77]	Beneficial effect of NPR-C inhibition
	Atherosclerosis (*ApoE⁻/⁻*) models	Global *Npr3* knockout Endothelial *Npr3* deletion	Decreased total aortic plaque burden, reduced vascular inflammation, and restricted endothelial apoptosis [78]	Beneficial effect of NPR-C deletion
	Murine model of vascular fibrosis	C-ANP_4−23_ treatment	Prevented pathological neoangiogenesis in fibrotic vascular tissue and downregulated VEGF expression [79]	Beneficial effect of NPR-C inhibition
	Murine model of aortic aneurysms	CNP stimulation in global *Npr3* knockout	Endothelial- and fibroblast-derived CNP signals through NPR-C to maintain aortic structural mechanics and blunt aneurysm expansion [80]	Beneficial effect of NPR-C activation
	Pulmonary vascular disease models	NPR-C agonist or *Npr3* knockout	Preserved pulmonary vascular homeostasis and cardiac performance under metabolic stress [81–83]	Context-dependent; both activation and deletion as protective
Liver	Murine liver in high-fat diet models	Musclin administration	Protected against the development of diet-induced hepatic steatosis across both sexes [71]	Beneficial effect of NPR-C inhibition
	Hepatocyte-specific models	*Npr3* overexpression or knockdown	Direct hepatocyte NPR-C expression actively accelerated steatosis through a clearance-independent USP30–C/EBPβ lipogenic program [84]	Harmful effect of NPR-C overexpression
	Experimental steatohepatitis models	CNP overexpression or analogue	Ameliorated liver steatosis, suppressed hepatic stellate cell activation/fibrosis, and lowered portal pressures [85]	Beneficial effect of NPR-C activation

*AF* Atrial fibrillation, *Ang II* Angiotensin II, *ANP* atrial natriuretic peptide, *ApoE⁻/⁻* apolipoprotein E–deficient, *C/EBPβ* CCAAT/enhancer-binding protein β, *CNP* C-type natriuretic peptide, *EDHF* endothelium-derived hyperpolarizing factor, *FSGS* focal segmental glomerulosclerosis, *G*_*i*_ inhibitory G protein, *HF* heart failure, *MI* myocardial infarction, *NaCl* sodium chloride, *NP* natriuretic peptide, *Npr3* gene encoding NPR-C, *NPR-C* natriuretic peptide receptor-C, *PKA* protein kinase A, *PKG* protein kinase G, *SGBS* Simpson-Golabi-Behmel Syndrome, *SHR* spontaneously hypertensive rats, *TGF-β* transforming growth factor-β, *USP30* ubiquitin-specific peptidase 30

### NPR-C in the adipose tissue and metabolism

As already stated above, in human obesity, the upregulation of NPR-C in adipose tissue serves as the primary anatomical driver of the systemic “natriuretic peptide handicap.” Visceral and subcutaneous adipose tissue biopsies from individuals with obesity reveal highly elevated NPR-C expression that correlates positively with the degree of adipocyte hypertrophy, fasting insulin levels, and the overall clinical severity of insulin resistance [28, 29]. From a genetic perspective, specific public variants within the human *NPR3* locus are associated with lower baseline adiposity measures and a reduced long-term risk of developing overweight or obesity, suggesting that variations in receptor density alter lifelong metabolic trajectories [86]. Conversely, evidence regarding its endogenous antagonist, musclin/osteocrin, remains largely observational in clinical cohorts. Elevated circulating concentrations of musclin are consistently tied to obesity, insulin resistance, type 2 diabetes mellitus (T2DM), and diabetic nephropathy [87–89]. In individuals diagnosed with metabolic syndrome, serum musclin levels correlate closely with hyperinsulinemia and adverse body composition metrics [89–91]. While these findings firmly link the musclin–NPR-C axis to human metabolic dysfunction, they remain associative and may represent either an active pathogenic driver or a compensatory, defensive response to chronic overnutrition.

The strongest functional and causal evidence for a direct metabolic regulatory role of NPR-C comes from genetically modified rodent models. Global *Npr3* knockout mice display a striking metabolic phenotype characterized by accelerated white adipose tissue (WAT) browning, markedly increased baseline energy expenditure, an improved overall lipid profile, and absolute resistance to diet-induced obesity [66]. To isolate the tissue-specific contribution of this pathway, investigators developed conditional deletion models. Adipose tissue-specific *Npr3* deletion attenuates chronic adipose inflammation, downregulates macrophage infiltration, and shifts adipocytes toward a highly oxidative, thermogenic phenotype in hypercholesterolemic mice [67]. Interestingly, skeletal muscle-specific *Npr3* deletion fails to alter exercise capacity or systemic glucose disposal, confirming that the primary metabolic actions of NPR-C are restricted to adipose compartments [68, 69].

Pharmacological interventions in animals further support this metabolic axis. In leptin-deficient (*ob/ob*) mice presenting with severe obesity and type 2 diabetes, chronic treatment with the selective NPR-C ligand C-ANP_4−23_ successfully minimizes total adiposity and restores peripheral insulin sensitivity by displacing endogenous NPs and reviving local signaling [73]. In obese, insulin-resistant rodents, musclin expression is upregulated by high-fat diets but downregulated by chronic exercise [89, 92–94]. Recombinant musclin administration or treatment with its bioactive peptide fragments similarly restricts visceral lipid accumulation and modulates insulin signaling cascades in experimental models [71]. Conversely, chronic administration of the dual NPR-A/NPR-B agonist CD-NP triggers a compensatory upregulation of adipose NPR-C expression in mice, ultimately worsening adiposity and metabolic parameters [95]. In murine models, CNP appears to regulate energy metabolism through both NPR-C-dependent and NPR-C-independent mechanisms, including suppression of sympathetic thermogenic activity via NPR-C/G_*i*_ signaling [96].

At the cellular level, the NPR-C expression is directly modulated by nutrient excess and hormonal signaling. In mature human adipocytes and murine 3T3-L1 systems, NPR-C mRNA and protein expression increase progressively during normal adipocyte differentiation. This expression is profoundly amplified following exposure to incremental, pathophysiological concentrations of glucose and insulin [30, 97]. This process is mediated via long-range distal enhancers where key adipogenic master regulators, including peroxisome proliferator-activated receptor-γ (PPARγ) and fatty acid binding protein 4 (FABP4), directly bind and remodel the *Npr3* promoter region during nutrient stress [97]. Furthermore, non-coding RNA networks have been identified as upstream epigenetic gatekeepers. Specifically, *miR-146a* acts as a direct post-transcriptional repressor of *Npr3* expression; an experimental loss of *miR-146a* drives rapid NPR-C upregulation, adipocyte hypertrophy, and a severe reduction in insulin-stimulated glucose uptake, whereas direct siRNA-mediated silencing of *Npr3* completely restores insulin-responsive glucose transport and normalizes de novo lipogenesis [52]. In isolated rat skeletal muscle cells, musclin administration actively interferes with metabolic homeostasis by directly inhibiting downstream insulin receptor/Akt signaling [70], whereas in 3T3-L1 adipocytes, it upregulates lipolytic pathways and suppresses lipogenesis via specific PKA/p38-MAPK mechanics [72].

### Cardiac disease

In the CKLM syndrome context, systemic drivers including obesity, insulin resistance, and chronic inflammation may precipitate a distinct pathological cardiac phenotype characterized by concentric remodeling, microvascular rarefaction, and interstitial fibrosis, culminating in diastolic impairment, HFpEF, and atrial fibrillation (AF) [98, 99].

Human observational data firmly link altered myocardial NPR-C expression to these failing states. Tissue analyses of explanted failing human hearts consistently reveal a significant upregulation of myocardial NPR-C expression [100, 101].

Furthermore, diabetes and impaired glucose tolerance independently upregulate cardiac *NPR3* transcription, illustrating a direct translational bridge between systemic metabolic stress and local cardiac receptor remodeling [102]. Large-scale human genetic studies have identified a highly prevalent, functional missense variant within the third exon of the human receptor (*N521D*) that is independently associated with the development of left ventricular diastolic dysfunction—the pathophysiological hallmark of human obesity-driven HFpEF [103].

This relationship is mirrored in preclinical models, where visceral adiposity, hypertension, and cardiac hypertrophy—phenotypes typical of human obesity-related HFpEF—correlates with NPR-C upregulation [104]. Causal evidence confirming that NPR-C directly dictates cardiac structural remodeling is heavily derived from animal models of pressure overload and ischemia. The endogenous musclin/osteocrin–NPR-C axis appears to be a defensive mechanism during cardiac stress. Because musclin binds competitively to NPR-C, it acts as a natural clearance inhibitor. In murine models of myocardial infarction (MI) and transverse aortic constriction (TAC), genetic overexpression or exogenous administration of musclin/osteocrin significantly dampens left ventricular dilation and delays the transition to overt HF [58, 59]. These systemic benefits are driven by the preservation of local atrial and BNP bioavailability, which directly suppresses cardiomyocyte hypertrophy, reduces reactive interstitial fibrosis, and protects contractility [58, 59].

However, global genetic deletion models expose a highly nuanced, dual role for this receptor. When subjected to chronic angiotensin II (Ang II) infusions to induce hypertensive heart disease, global NPR-C^−/−^ mice show a significantly more severe decline in ejection fraction, profound sinoatrial node dysfunction, and rapid decompensation into overt, dilated heart failure compared to wild-type controls [53]. Similarly, the atria of Ang II-treated NPR-C^−/−^ mice exhibit a massive structural fibrosis burden and a stark increase in inducible AF [54]. Crucially, cotreating wild-type mice with Ang II and the selective ligand C-ANP_4−23_ successfully prevents these electrical and structural abnormalities, dose-dependently reducing AF susceptibility [54]. In sharp contrast, within rodent models of diabetic cardiomyopathy, global or cardiac-specific *Npr3* knockout yields an entirely beneficial phenotype, significantly reducing myocardial fibrosis and maintaining normal ventricular relaxation independently of circulating systemic NP levels [56]. In the setting of acute induced cardiac ischemia, the administration of C-ANP_4−23_ during the reperfusion phase alone provides intense cardioprotection, successfully promoting coronary vasodilation, limiting infarct size, and preserving left ventricular developed pressures [60].

 In vitro cellular models reveal that NPR-C acts via distinct, clearance-independent intracellular signaling pathways within cardiac cells. In isolated rodent cardiomyocyte and sinoatrial node preparations, ligand binding to NPR-C modulates cardiac chronotropy and dromotropy via a G_*i*_-protein-dependent pathway. This signaling directly inhibits adenylyl cyclase, suppresses intracellular cAMP generation, and dynamically alters L-type calcium and hyperpolarization-activated cyclic nucleotide-gated (HCN) ion channel kinetics [105].

Within isolated human and rodent cardiac fibroblasts, NPR-C acts as an active signaling node driving anti-remodeling responses. Deletion or targeted pharmacological antagonism of NPR-C in diabetic cardiac fibroblasts completely blocks the anti-fibrotic actions of BNP, an effect found to be independent of cGMP. Instead, excessive NPR-C activation under diabetic conditions actively downregulates protective protein kinase A and G (PKA/PKG) intracellular signaling while cross-activating transforming growth factor-beta 1 (TGF-β1)/Smad transcription programs, directly driving the fibroblast-to-myofibroblast transformation and the extracellular matrix deposition [55–57]. Furthermore, cardiomyocyte-derived CNP utilizes local endothelial NPR-C signaling to activate local pro-angiogenic pathways, maximizing capillary revascularization following hypoxic injury [61].

### Renal disease

Metabolic kidney disease within the CKLM framework is driven by glomerular hypertension, hyperfiltration, and podocyte injury—phenotypes often exacerbated by systemic hypertension, RAAS overactivation, and dyslipidemia [106, 107].

Historically confined to animal systems, recent high-impact translational evaluations utilizing human renal biopsy tissue have successfully validated the presence of this pathway in clinical disease. Quantitative transcriptomic and immunohistochemical profiling of renal biopsies from patients with advanced diabetic kidney disease (DKD) and focal segmental glomerulosclerosis (FSGS) revealed a pronounced upregulation of NPR-C localized heavily within the glomerular filtration barrier and podocyte cytoplasm [62]. The functional consequences of renal NPR-C overactivation have been dissected using sophisticated cell-specific genetic models. The role of this receptor was demonstrated using podocyte-specific *Npr3* knockout mice. When subjected to experimental diabetic conditions, mice lacking podocyte NPR-C display near-complete protection against metabolic renal injury, showing a profound reduction in albuminuria, preservation of podocyte foot process architecture, and a complete suppression of intra-renal pro-inflammatory and pro-fibrotic gene expressions [62, 63]. Strikingly, this protective phenotype occurs independently of changes in systemic, circulating ANP or BNP levels, proving that podocyte NPR-C actively mediates local cellular injury rather than operating as a passive systemic clearance sink [62, 63].

These findings are complemented by pharmacological studies in proteinuric models. In murine configurations of FSGS and hypertensive kidney injury, direct administration of the selective ligand C-ANP_4−23_ significantly attenuates proteinuria, reduces structural glomerulosclerosis, and dampens aldosterone-mediated mineralocorticoid receptor overactivation [49, 64]. Conversely, findings from global *Npr3* knockout models have shown less consistency in acute glomerulonephritis settings due to systemic hemodynamic shifts, pointing to the absolute necessity of tissue-targeted interventions [108]. Additionally, targeted deletion of NPR-C within isolated distal tubule cells successfully dampens the hyper-activation of the NaCl cotransporter, blunting Ang II-dependent systemic hypertension [65].

Mechanistic basic science studies have mapped the precise intracellular signaling cascades through which podocyte NPR-C drives filtration barrier breakdown. In cultured podocytes exposed to high-glucose or advanced glycation end-products, overexpressed NPR-C physically associates with intracellular trafficking machinery. Specifically, NPR-C activation triggers a clearance-independent, endosomal recycling pathway mediated by Rab6a and Rab8a small GTPases. This pathway prevents the normal degradation of the TGF-β receptor type II (TGF-βR2), routing it back to the cell surface [62]. The resulting surface accumulation of TGF-βR2 hyper-sensitizes the podocyte to low levels of local ambient TGF-β, driving downstream Smad2/3 phosphorylation, actin cytoskeleton reorganization, nephrin downregulation, and eventual podocyte apoptosis [62, 63]. Concurrently, overactive tubular NPR-C suppresses local adenylyl cyclase activity, reducing intracellular cAMP concentrations and diminishing the renal tubuloglomerular feedback mechanism required to offset glomerular hyperfiltration, in contrast to the renoprotective effects of sodium-glucose cotransporter 2 inhibitors (SGLT2i) [109].

### Hypertension and vascular disease

Vascular disease in CKLM encompasses systemic hypertension, macrovascular atherosclerosis, microvascular rarefaction, endothelial dysfunction, and pulmonary vascular remodeling [110, 111]. NPR-C is widely expressed throughout the vascular network, localized predominantly within endothelial cells and vascular smooth muscle cells (VSMCs) [112, 113]. Current evidence suggests a complex, dual-function model where NPR-C acts both as a clearance receptor that limits protective NP bioavailability and as a signaling receptor mediating tissue-specific, G_*i*_-dependent pathways [38]. The balance between these two functions, together with the vascular bed and disease context under investigation, likely determines the net physiological outcome.

#### Large arteries and systemic hypertension: detrimental roles of excessive clearance

In large systemic arteries exposed to mechanical and metabolic stress, excessive NPR-C activity typically correlates with accelerated vascular injury. In human genetic studies, specific *NPR3* polymorphisms have been independently associated with an increased risk of coronary atherosclerosis, persisting even after adjustment for conventional risk factors such as age, sex, smoking, hypertension, diabetes, and dyslipidemia [114]. This genetic predisposition matches histological data from human tissue, where myocardial and vascular NPR-C expression correlates positively with the clinical severity of atherosclerotic lesions and features of advanced plaque vulnerability [115, 116]. Furthermore, human genetic evidence shows that reduced NPR-C function lowers the risk of preeclampsia—a condition sharing core CKLM features like acute endothelial dysfunction and systemic hypertension—reinforcing the hypothesis that overactive NPR-C drives vascular pathology by inducing local NP deficiency [117]. This paradigm is supported by animal models of systemic hypertension and atherogenesis. In several rodent models of hypertension, either pharmacological blockade or genetic knockdown of NPR-C enhances endogenous NP signaling and successfully lowers BP, confirming that receptor-mediated clearance actively drives vascular dysfunction by restricting access to the vasodilatory NPR-A pathway [48, 74, 76]. Similarly, targeted deletion of NPR-C in the distal nephron attenuates angiotensin II-induced hypertension via modulation of the NaCl cotransporter, pointing to direct detrimental crosstalk with the RAAS [65].

In atheroprone mice, endothelial-specific *Npr3* deletion significantly reduces both overall atherosclerotic plaque burden and markers of plaque vulnerability [79]. This is mirrored in experimental models of vascular fibrosis, where NPR-C antagonism suppresses pathological neovascularization and attenuates structural remodeling, downregulating vascular endothelial growth factor (VEGF) [78].

At the cellular level, the detrimental impacts of this axis are primarily tied to proliferative pathways. In isolated cell systems, the selective NPR-C ligand C-ANP_4−23_ has been shown to modulate VSMC behaviour directly. Depending on the cellular environment, it can trigger hyperproliferative responses or, conversely, inhibit smooth muscle cell proliferation through G_*i*_-protein-mediated activation of the MAP kinase and phosphatidylinositol 3-kinase (PI3K)/AKT signaling pathways, demonstrating that clearance-independent signaling operates directly within the vessel wall [77].

#### Microvasculature and pulmonary beds: protective roles of active signaling

In contrast to its maladaptive clearance role in the large vessels, intact NPR-C signaling appears essential for maintaining homeostasis within endothelial, microvascular, and pulmonary vascular beds. In murine models of systemic inflammation and sepsis, global *Npr3* deletion significantly worsens microvascular perfusion, exacerbates endothelial dysfunction, and accelerates end-organ injury [118]. The tight phenotypic overlap observed between *Npr3*-deficient mice and endothelial *Cnp*-deficient mice indicates that a protective, local CNP-NPR-C signaling axis is required to preserve microvascular patency and limit leukocyte adhesion during acute inflammation [38, 118].

Furthermore, in experimental models of pulmonary vascular injury and obesity-related lung disease, the loss of NPR-C worsens pulmonary hypertension and tissue damage, suggesting that intact receptor signaling protects the low-pressure pulmonary circulation against pathological remodeling [81–83, 119]. Mechanistic cellular studies confirm that endothelial-derived CNP acts locally via NPR-C to drive protective, localized autocrine and paracrine responses. Within the endothelium, ligand binding to the short intracellular domain of NPR-C couples directly to inhibitory G_*i*_ proteins. This interaction suppresses adenylyl cyclase activity but simultaneously stimulates endothelial nitric oxide synthase (eNOS) and activates endothelium-derived hyperpolarizing factor (EDHF) pathways [38, 120]. These downstream signals promote localized vasodilation, inhibit leukocyte transmigration, and protect against aortic aneurysm formation in vitro, providing a biochemical basis for the receptor’s protective microvascular actions [40, 80, 121–123].

Collectively, these data highlight that the net vascular impact of NPR-C is highly tissue- and context-dependent. While human genetic and clinical data firmly align with animal models in identifying macrovascular NPR-C clearance as a detrimental driver of hypertension and plaque vulnerability, the proposed protective microvascular signaling pathways remain entirely confined to preclinical rodent and cell models. Translational validation in human microvascular kinetics is required to confirm this dual-action framework.

### Hepatic disease

As the newest cornerstone of the CKLM paradigm, metabolic dysfunction–associated steatotic liver disease (MASLD) and its progressive inflammatory phenotype, metabolic dysfunction–associated steatohepatitis (MASH), represent a direct hepatic reflection of metabolic syndrome [124, 125].

Clinical and epidemiological data regarding direct hepatic NPR-C expression remain preliminary, but human systemic imaging cohorts provide a clear rational framework. Large-scale multi-ethnic clinical investigations utilizing computed tomography (CT) to quantify hepatic steatosis demonstrate that absolute liver fat volume is robustly and inversely correlated with circulating NP fractions [126, 127]. This clinical observation indicates that systemic NP deficiency directly parallels ectopic hepatic lipid deposition.

This systemic relationship is robustly recapitulated in animal models of overnutrition. In mice subjected to aggressive high-fat diets, the systemic restoration of NP signaling—achieved either via direct exogenous chronic BNP infusions or through the global genetic deletion of the *Npr3* gene—confers a powerful hepatoprotective effect [126, 127].

Animals lacking functional NPR-C display a total reduction in ectopic hepatic lipid accumulation, an effect driven by enhanced systemic lipolysis, accelerated skeletal muscle fatty acid oxidation, and the rapid induction of energy-expending beige adipose tissue browning [126, 127]. Thus, macrovascular and adipose NPR-C expression indirectly regulates the initial stages of hepatic steatosis by governing whole-body lipid flux and preventing lipotoxicity.

Beyond these indirect systemic metabolic actions, emerging preclinical evidence confirms that NPR-C operates as a direct, intra-hepatic pathogenic driver within liver tissue. In animal models of diet-induced steatohepatitis, advanced hepatic profiling has localized significant NPR-C expression directly within hepatocytes, liver sinusoidal endothelial cells (LSECs), and quiescent hepatic stellate cells (HSCs) [84].

To evaluate the intra-hepatic therapeutic potential of this network, pharmacological studies utilizing targeted ligands have been deployed. In experimental models of severe steatohepatitis and established portal hypertension, the administration of selective long-acting CNP analogues or the targeted delivery of recombinant musclin significantly attenuates the structural progression of liver disease [71, 85]. These interventions achieve a marked reduction in total liver hydroxyproline content, minimize inflammatory cell infiltration, and lower portal pressures by restoring normal microcirculatory relaxation and blunting the activated, contractible phenotype of HSCs [85].

At the cellular and molecular level, recent basic science investigations have uncovered a direct, clearance-independent signaling cascade through which hepatocyte NPR-C actively commands cellular lipid synthesis. Utilizing isolated primary hepatocyte cultures, investigators demonstrated that overexpressed NPR-C binds directly to the deubiquitinating enzyme ubiquitin-specific peptidase 30 (USP30) within the cytoplasm [84]. Upon ligand activation, the intracellular domain of NPR-C triggers a signaling cascade that promotes USP30-mediated stabilization and deubiquitination of CCAAT/enhancer-binding protein beta (C/EBPβ), a major lipogenic transcription factor [84].

The resulting accumulation of stabilized C/EBPβ shifts the hepatocyte genome toward an aggressive lipogenic program, directly upregulating the transcription of key fatty acid synthesis enzymes and accelerating intracellular triglyceride assembly and vesicular steatosis independently of external NP concentrations [84]. Conversely, siRNA-mediated knockdown of hepatocyte *Npr3* completely disrupts this USP30–C/EBPβ axis, fully protecting the cell from oleic acid-induced lipid loading [84]. This cell-autonomous mechanism operates in parallel with localized G_*i*_-protein pathways within adjacent hepatic stellate cells, where overactive NPR-C suppresses the anti-fibrotic cAMP/PKA cascade, thereby promoting collagen synthesis and accelerating the transition from simple steatosis to irreversible inflammatory fibrosis [85].

## Global summary of systemic and organ actions

**Fig. 2:**
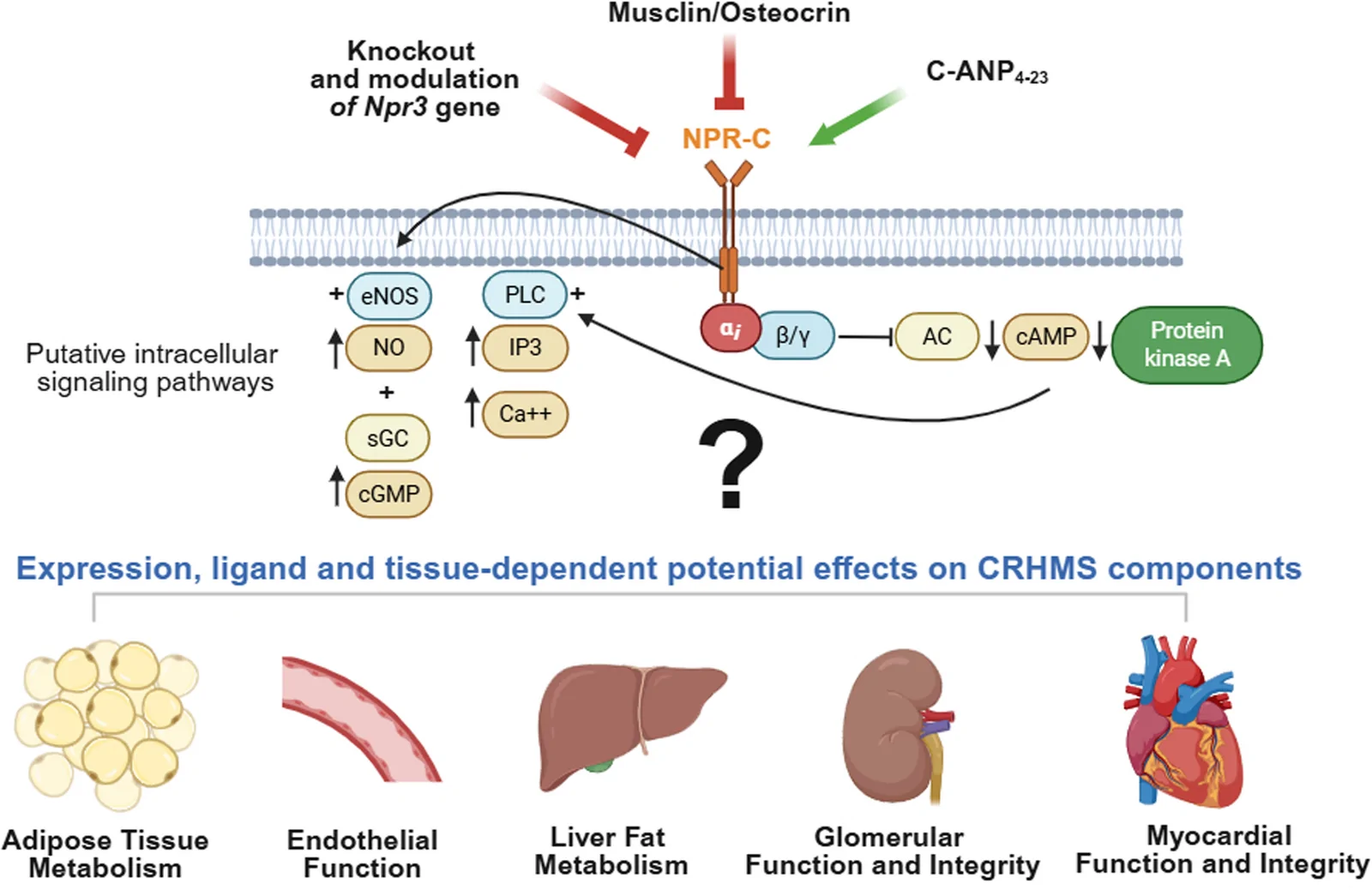
Potential tissue-specific effects of direct NPR-C modulation. This figure outlines the putative intracellular signaling of NPR-C and the potential systemic consequences of its modulation. Unlike the guanylate cyclase-linked receptors, NPR-C couples with G_*i*_ proteins, leading to the inhibition of the adenylate cyclase (AC) and cAMP/Protein Kinase A (PKA) pathway, increased phospholipase C (PLC)/inositol triphosphate (IP3)/Ca^++^ signaling and endothelial nitroxide synthase (eNOS)/nitroxide (NO) activity. The diagram highlights how the genetic or ligand-mediated modulation of the *Npr3* gene and NPR-C can shift this balance. These modulations result in potential tissue-specific effects across multiple systems, including adipose tissue, vascular endothelium, liver, kidney glomeruli, and myocardium

Collectively, these experimental data underpin an extended and diversified role of NPR-C within the CKLM framework. While its well described clearance capacity remains fundamental, here we highlighted the potential direct regulatory influence on pathological hallmarks of disease progression and tissue remodeling. We propose a multifaceted model of NPR-C activity—transitioning from passive scavenger to active therapeutic target—as depicted in Fig. 2.

## Gaps in knowledge and future directions

### Human validation of NPR-C signaling beyond peptide clearance

Despite substantial advances in understanding NPR-C biology, the evidence base remains heavily weighted toward experimental studies. In humans, the most consistent finding is the association between increased adipose NPR-C expression and reduced NP bioavailability in obesity, supporting the concept of NPR-C as a major clearance receptor. By contrast, evidence supporting direct NPR-C-mediated intracellular signaling remains largely confined to in vitro systems and animal models. Although multiple pathways involving G*i* proteins, TGF-β signaling, inflammatory mediators, and metabolic regulators have been described, their physiological relevance in humans remains uncertain. Consequently, a major research priority is to determine whether NPR-C functions as a biologically relevant signal transducer in human disease or whether its predominant contribution to CKLM pathology derives from modulation of NP availability.

### Tissue-specific and ligand-specific NPR-C signaling

A recurring challenge across cardiovascular, renal, hepatic, and metabolic studies is the apparent context dependency of NPR-C biology. In some experimental settings, NPR-C deletion or antagonism appears beneficial by enhancing NP bioavailability and reducing fibrosis, inflammation, or metabolic dysfunction. Conversely, activation of NPR-C by endogenous ligands such as CNP has been shown to exert protective effects, particularly within endothelial, pulmonary, and microvascular compartments. These observations suggest that NPR-C may exert distinct biological actions depending on the tissue, disease state, and ligand involved. Future studies should clarify the relative contribution of NPR-C-mediated clearance and direct signaling, as well as determine whether ligand-specific or “biased” signaling mechanisms exist in human physiology.

### The musclin/osteocrin–NPR-C axis

The musclin/osteocrin–NPR-C axis represents one of the most intriguing yet controversial areas of NPR-C research. Experimental evidence suggests that musclin and osteocrin competitively inhibit NPR-C-mediated NP clearance, thereby augmenting endogenous NP signaling and promoting cardiometabolic protection. However, conflicting findings have emerged regarding blood pressure regulation, with some animal studies reporting antihypertensive effects and others demonstrating paradoxical pressor responses [75]. These discrepancies may reflect tissue-specific actions, differential ligand competition, compensation mechanisms or direct signaling effects independent of NP clearance. Clarifying the physiological and pathological significance of musclin/osteocrin signaling in humans remains an important unmet need.

### Sex as a biological variable in NPR-C research

Sex-related differences are well established within the broader NP system. Women generally exhibit higher circulating NP concentrations than men, and differences in peptide processing, clearance, and plasma half-life have been described [128, 129]. These observations likely reflect interactions among sex hormones, adipose tissue distribution, and body composition. However, little is known regarding sex-specific regulation of NPR-C expression or function. Significantly higher renal and mesenteric adipocyte NPR-C expression in overweight, estrogen-deficient models was described as reversible by hormonal treatment [130], further cementing the tight mechanistical relationship between estrogen deficit, adipose tissue accumulation, and dysregulated NPR-C signaling. The demonstration of sexual dimorphism within other NP pathways, such as the CNP–NPR-B axis, raises the possibility that NPR-C biology may also be influenced by biological sex [131]. Future experimental and clinical studies should therefore incorporate sex-specific analyses to determine whether NPR-C contributes to differences in cardiometabolic disease susceptibility, NP bioavailability, and therapeutic responsiveness.

### Interaction with contemporary CKLM therapies

The emergence of therapies with proven cardiovascular, renal, hepatic, and metabolic benefits has transformed the management of CKLM-related disorders. However, their interaction with NPR-C biology remains poorly understood. Angiotensin receptor–neprilysin inhibitors (ARNIs) provide an important proof-of-concept that augmenting NP bioavailability can improve outcomes across multiple organ systems [132–136]. Beyond their established role in heart failure, ARNIs improve insulin sensitivity, promote lipolysis and lipid oxidation, and induce favorable adipose tissue remodeling, supporting the concept that enhancement of NP signaling may confer broad cardiometabolic benefits [137–139].

Nevertheless, whether these effects intersect with NPR-C-dependent pathways remains unknown. Similarly, little information is available regarding the impact of sodium-glucose cotransporter-2 inhibitors (SGLT2i), glucagon-like peptide-1 receptor agonists (GLP-1RA), or non-steroidal mineralocorticoid receptor antagonists on NPR-C expression, function, or downstream signaling. Understanding these interactions may identify opportunities for therapeutic synergy and help define the position of NPR-C-targeted therapies within the evolving CKLM treatment landscape.

### Development of NPR-C-targeted therapies

Given the dual biological role of NPR-C as both a clearance receptor and a signaling receptor, several distinct therapeutic strategies can be envisioned, each with potentially different biological and clinical consequences. The most immediate and biologically plausible approach is the modulation of NPR-C-mediated NP clearance, either through competitive ligands (e.g., musclin/osteocrin-derived compounds) or selective NPR-C antagonists designed to increase endogenous NP bioavailability. Conceptually, this strategy parallels the enhancement of NP signaling achieved through neprilysin inhibition, albeit by targeting a complementary regulatory pathway. A second, more speculative approach could involve selective modulation of NPR-C intracellular signaling. The emerging experimental evidence suggesting that NPR-C may exert tissue-specific profibrotic, proinflammatory, or protective effects independent of peptide clearance, raises the possibility that biased ligands could selectively activate or inhibit distinct downstream pathways to achieve organ-specific therapeutic benefits. Finally, indirect modulation of NPR-C expression through genetic, epigenetic, or microRNA-based approaches has shown promising results in preclinical studies, although these strategies remain far from clinical application.

Consistent with these concepts, several NPR-C-targeted interventions, including microRNA-based therapies, peptide ligands, antibodies and small-molecule modulators, have demonstrated favorable effects on metabolic dysfunction, fibrosis, inflammation, and organ remodeling in experimental models [50, 51, 140]. However, the available evidence remains largely preclinical, and none of these approaches has yet progressed to advanced clinical testing. Consequently, their long-term safety, tissue specificity, and therapeutic efficacy in humans remain unknown. Notably, a long-acting, once-weekly subcutaneous CNP analogue that has shown encouraging hemodynamic effects in hypertensive animal models is expected to enter phase I clinical evaluation in patients with HFpEF, representing one of the first translational efforts to therapeutically modulate this pathway [141].

An important challenge for future drug development stems from the context-dependent nature of NPR-C biology. Because NPR-C regulates both NP clearance and intracellular signaling, indiscriminate receptor activation or inhibition may produce divergent—and potentially opposing—effects across different tissues and disease states. Therefore, the development of selective modulators capable of targeting specific NPR-C functions, signaling pathways, or tissue compartments will likely be necessary to fully harness the therapeutic potential of this receptor within the CKLM spectrum.

### Beyond the natriuretic peptide deficiency paradigm

Current human evidence largely supports the concept that NPR-C contributes to CKLM through enhanced NP clearance and the resulting relative NP deficiency observed in obesity and metabolic dysfunction. However, emerging experimental data suggest that this paradigm may be incomplete. A more comprehensive model may need to integrate NPR-C-mediated peptide clearance, ligand competition, direct intracellular signaling, and tissue-specific receptor functions. Elucidating how these mechanisms interact across the cardiovascular, renal, hepatic, and metabolic continuum will be essential for determining whether NPR-C represents merely a biomarker of disease severity or a true therapeutic target capable of modifying the natural history of CKLM syndrome.

## Data Availability

No datasets were generated or analysed during the current study.

## Abbreviations

AF: Atrial fibrillation
ANP: Atrial natriuretic peptide
ApoE: Apolipoprotein E
ARNIs: Angiotensin receptor–neprilysin inhibitors
BNP: B-type natriuretic peptide
BP: Blood pressure
C/EBPβ: CCAAT/enhancer-binding protein beta
cGMP: Cyclic guanosine monophosphate
CKD: Chronic kidney disease
CKLM: Cardiovascular-renal-hepatic-metabolic syndrome
CNP: C-type natriuretic peptide
eNOS: Endothelial nitric oxide synthase
EDHF: Endothelium-derived hyperpolarizing factor
FABP4: Fatty acid binding protein 4
FSGS: Focal segmental glomerulosclerosis
G_*i*_: Inhibitory G protein
HF: Heart failure
HSCs: Hepatic stellate cells
LSECs: Liver sinusoidal endothelial cells
MASLD: Metabolic dysfunction–associated steatotic liver disease
MASH: Metabolic dysfunction–associated steatohepatitis
MI: Myocardial infarction
NP: Natriuretic peptide
NPR-A: Natriuretic peptide receptor A
NPR-B: Natriuretic peptide receptor B
NPR-C: Natriuretic peptide receptor C
*Npr3*: Natriuretic peptide receptor 3 gene (animal)
*NPR3*: Natriuretic peptide receptor 3 gene (human)
PKA: Protein kinase A
PKG: Protein kinase G
PLC: Phospholipase C
PPARγ: Peroxisome proliferator-activated receptor-γ
RAAS: Renin-angiotensin-aldosterone system
SGLT2: Sodium-glucose cotransporter 2
SHR: Spontaneously hypertensive rats
T2DM: Type 2 diabetes mellitus
TGF-β: Transforming growth factor-β
USP-30: Ubiquitin-specific peptidase 30
VEGF: Vascular endothelial growth factor
VSMCs: Vascular smooth muscle cells
